# Optimized Plasmonic Gold-Pillar Metasurfaces for Refractive-Index Sensing

**DOI:** 10.3390/nano16140841

**Published:** 2026-07-09

**Authors:** Liliana Valente, Dante M. Aceti, Rossella Zaffino, Giovanna Palermo, Giuseppe Strangi

**Affiliations:** 1 NLHT-Lab, Department of Physics, University of Calabria and CNR-NANOTEC, Institute of Nanotechnology, 87036 Rende, Italy; liliana.valente@unical.it (L.V.); dante_maria.aceti@unical.it (D.M.A.); gxs284@case.edu (G.S.); 2 ICFO—Institut de Ciencies Fotoniques, The Barcelona Institute of Science and Technology, 08860 Castelldefels, Spain; rossella.zaffino@icfo.eu; 3 Department of Physics, Case Western Reserve University, 2076 Adelbert Rd, Cleveland, OH 44106, USA

**Keywords:** metasurfaces, plasmonics, sensing, near-field enhancement, refractive-index sensing

## Abstract

Gold pillar-based plasmonic metasurfaces provide a robust platform for optical sensing owing to their strong localized surface plasmon resonances and tunable near-field distributions. In this work, we present a numerical investigation of a gold-pillar metasurface optimized specifically for high-performance refractive-index sensing. A comprehensive parametric optimization of the metasurface was carried out, including analyses of the inter-pillar gap, the thickness of the underlying gold film, and the optical response under varying angles of incidence. Numerical analysis demonstrates that the engineered plasmonic pillar array achieves a sensitivity of 500 nm/RIU and a Figure of Merit (FOM) of 79. These results demonstrate the potential of the proposed plasmonic metasurface as an optimized platform for high-performance refractive-index sensing, providing practical design guidelines for future chemical and biological sensing applications.

## 1. Introduction

The engineering of light–matter interactions at the subwavelength scale has led to the development of metamaterials and, more recently, metasurfaces—ultra-thin, planar, and engineered structures composed of subwavelength resonant elements (“meta-atoms”) designed to provide precise control over amplitude, phase, and polarization of light [1,2,3]. Recent advances in metasurface photonics have demonstrated that optical performance can be substantially enhanced through alternative resonance-engineering strategies, including topologically protected quasi-bound states in the continuum (quasi-BICs), nonlocal metasurfaces, continuously tunable three-dimensional metastructures, and topologically engineered multifunctional platforms [4,5,6,7]. These approaches exploit high-Q resonances, enhanced near-field confinement, advanced dispersion engineering, and multidimensional control of light to achieve unprecedented optical functionalities. For example, topologically engineered quasi-BIC metasurfaces have demonstrated remarkable near-field enhancement and ultrahigh-Q resonances for enhanced light–matter interaction [4], while nonlocal metasurfaces have enabled continuous polarization–wavelength mapping through advanced dispersion engineering [6]. Furthermore, continuously tunable three-dimensional metastructures have shown the potential of refractive-index-mediated spectral control [5], and topologically engineered Möbius metasurfaces have demonstrated fully decoupled manipulation of multiple optical degrees of freedom [7]. Although primarily developed for applications such as light emission enhancement, multidimensional wavefront manipulation, optical encryption, and advanced photonic information processing, these studies clearly highlight the importance of resonance engineering and structural optimization in modern metasurface design. Motivated by these developments, the present work investigates a complementary plasmonic approach based on the optimization of a hybrid Au nanopillar–Au film architecture supporting coupled localized surface plasmon resonance (LSPR) and surface plasmon polariton (SPP) modes for high-performance refractive-index sensing. From an electromagnetic standpoint, the behavior of metasurfaces is governed by Maxwell’s equations, together with the constitutive relations [8]. Among the various classes, plasmonic metasurfaces exploit metallic nanostructures to support localized surface plasmon resonances (LSPRs) and, in the presence of continuous metallic films, surface plasmon polaritons (SPPs) [9,10,11,12].

These resonances are characterized by strong electromagnetic field confinement near the metal surface, leading to enhanced light–matter interactions [11,13].

Besides wavefront engineering, plasmonic metasurfaces have also emerged as versatile platforms for enhancing light–matter interactions through collective plasmonic resonances, enabling applications in spectroscopy, nonlinear optics, and refractive-index sensing. In these systems, the emphasis is placed on tailoring the resonant optical response rather than exclusively controlling the phase of transmitted or reflected waves. By carefully tailoring the geometry and arrangement of these elements, metasurfaces can modulate the interaction between electromagnetic waves and matter in ways that are unattainable with conventional optics [14,15]. Compared to volumetric metamaterials, metasurfaces offer significant advantages in terms of reduced thickness, lower optical losses, and compatibility with standard nanofabrication techniques, making them particularly attractive for advanced photonic applications such as flat optics and optical sensing [2,3,15].

Owing to this property, plasmonic metasurfaces constitute highly effective platforms for refractive index sensing, as even small perturbations in the surrounding dielectric environment can induce measurable spectral shifts of the optical resonances [11,16,17,18,19].

The sensitivity of a plasmonic sensor is commonly defined as S = $\Delta \lambda_{res}$/Δn, where $\Delta \lambda_{res}$ is the shift in resonance wavelength induced by a refractive index variation Δn [11]. However, a more comprehensive evaluation of sensor performance requires the introduction of the figure of merit (FOM), FOM = S/FWHM, which also accounts for the spectral linewidth (full width at half maximum, FWHM) of the resonance and thus reflects both sensitivity and spectral resolution [20,21].

The simultaneous optimization of sensitivity, FOM, and near-field confinement represents a central objective in the design of plasmonic metasurfaces for sensing applications [17]. The coexistence of LSPR- and SPP-mediated resonances enables sharp spectral features and strong electromagnetic field confinement at both the edges of nanostructures and in the vicinity of continuous metallic films, ensuring efficient interaction with analytes deposited on or near the surface [12,22]. This characteristic is especially important in biosensing scenarios, where target analytes often form ultrathin molecular layers [23,24].

Recent advances in plasmonic metasurfaces have shown that coupling metallic nanopillars with continuous metallic films enables the excitation of hybrid plasmonic modes combining LSPRs and propagating SPPs [25,26]. Such hybrid resonances can simultaneously provide strong electromagnetic near-field confinement and spectrally sharp optical features, both of which are highly desirable for refractive-index sensing applications. In particular, pillar-on-film architectures offer enhanced electromagnetic coupling and increased interaction volumes compared to isolated nanoparticle systems, enabling improved sensitivity toward ultrathin dielectric perturbations and molecular adsorption events. Recent studies have demonstrated that periodic plasmonic arrays exploiting surface lattice resonances (SLRs) or Wood-anomaly-assisted plasmonic coupling can achieve exceptionally narrow linewidths and figures of merit exceeding 100 by combining localized plasmon resonances with collective diffraction effects. In particular, Li et al. demonstrated reflection-type SLR metasurfaces for high-performance refractive-index sensing, while Shen et al. reported gold mushroom arrays operating through the interference between localized plasmons and Wood’s anomalies, approaching the theoretical FOM limit for plasmonic sensors [19,27]. These studies highlight the potential of collective resonant phenomena for optical sensing, while also emphasizing that different structural configurations exploit distinct physical mechanisms to optimize sensing performance. Nevertheless, achieving the simultaneous optimization of resonance linewidth, spectral contrast, field confinement, and refractive-index sensitivity remains a significant challenge, as the optical response strongly depends on geometrical parameters such as inter-pillar spacing, metallic film thickness, and illumination conditions.

In this context, the present work focuses on the systematic optimization of a gold pillar-on-film plasmonic metasurface operating in the visible spectral range for hybrid plasmonic refractive-index sensing. Unlike conventional plasmonic nanoparticle arrays, the proposed architecture supports the coexistence of localized and propagating plasmonic modes, enabling the generation of multiple high-contrast resonances with distinct near-field distributions. By combining far-field spectral analysis with near-field electromagnetic mapping, we investigate how inter-pillar coupling, film thickness, and excitation geometry influence the spectral response and sensing performance of the metasurface. The optical response of the investigated metasurface was numerically analyzed using COMSOL Multiphysics 6.2 by solving Maxwell’s equations in the frequency domain through the finite element method (FEM). The simulations were specifically designed to accurately reproduce the electromagnetic response of periodic plasmonic nanostructures, including LSPRs, SPP excitation, and near-field coupling effects between adjacent metallic elements.

## 2. Materials and Methods

The numerical simulations were performed using COMSOL Multiphysics 6.2, solving Maxwell’s equations in the frequency domain through the Finite Element Method (FEM) implemented in the Electromagnetic Waves, Frequency Domain (EWFD) module [28,29]. The frequency-domain approach allows the description of the propagation of harmonic electromagnetic waves in linear, isotropic, and dispersive media, and is particularly well suited for investigating light–matter interactions in plasmonic nanostructures and metasurfaces. The modeled system consists of a three-dimensional unit cell representative of the periodic plasmonic metasurface, comprising the nanostructure and a gold layer, deposited on a dielectric substrate and embedded in a homogeneous surrounding medium. Infinite periodicity in the transverse directions was reproduced through the Periodic Structure feature of the RF Module, which automatically combines Floquet-periodic boundary conditions on the lateral boundaries with periodic excitation ports along the propagation direction. Consequently, Floquet-periodic boundary conditions were imposed along the *x*- and *y*-directions to emulate an infinite array of periodically repeated unit cells.

Along the propagation direction (*z*) two electromagnetic ports were defined, one acting as an input port, and the other as an output port, for excitation and collection of the transmitted and reflected signals, respectively [28,29,30]. To simulate open-space conditions and suppress spurious reflections at the boundaries of the computational domain, Perfectly Matched Layers (PMLs) were introduced at the upper and lower ends of the geometry. The illumination was modeled as an incident plane wave impinging at an angle θ with respect to the surface normal, appropriately selected according to the optical phenomenon under investigation, by means of an input port for the emitted radiation (Port 1) and an output port for detection (Port 2). To accurately reproduce free-space propagation, the computational domain included homogeneous media extending for a distance of λ above the metasurface and λ below the substrate, where λ denotes the excitation wavelength. At both ends of the computational domain, Perfectly Matched Layers (PMLs) with a thickness of 0.25λ were introduced to suppress spurious reflections from the external boundaries and mimic open-boundary conditions. The electromagnetic excitation was considered linearly polarized, with the electric field oriented parallel to one in-plane direction of the metasurface. A physics-controlled tetrahedral mesh with Extremely Fine element size was employed throughout the computational domain. Automatic local refinement was generated at the gold nanopillar edges, metal–dielectric interfaces, and in the narrow regions supporting strong plasmonic field localization, while identical meshes were enforced on opposite periodic boundaries to ensure the correct implementation of the Floquet-periodic conditions. Mesh convergence was verified by progressively refining the discretization until negligible variations in the resonance wavelengths and reflectance spectra were observed.

In the harmonic regime, the complex electric field E(x,y,z) is obtained by solving the following vector wave equation:

(1) $$\nabla \times \left(\mu_{r}^{-1}\nabla \times E\right)-k_{0}^{2}\left(\varepsilon_{r}-\frac{j\sigma }{\omega \varepsilon_{0}}\right)E=0,$$

where $\varepsilon_{0}$ is the permittivity of free space, $\mu_{r}$ is the relative permeability, $\varepsilon_{r}$ is the complex relative permittivity, σ is the electrical conductivity, ω is the angular frequency, and $k_{0}$ is the free-space wavenumber.

Each unit cell of the metasurface consisted of three vertically stacked regions. At the bottom, a glass substrate with refractive index $n_{glass}=$1.5 supported the structure. Above the substrate, the metasurface consisted of a cylindrical gold nanopillar supported by a continuous gold film. The dispersive optical response of gold was modeled using the experimentally measured refractive index reported by Johnson and Christy [31]. The entire structure was finally embedded in a homogeneous surrounding medium with a variable refractive index $n_{medium}$, enabling the refractive index sensing [32].

The pillar-on-film configuration was selected because it enables simultaneous excitation of localized plasmonic modes supported by the nanopillars and propagating plasmonic modes sustained by the continuous metallic film. The electromagnetic coupling between these resonances produces hybrid optical modes characterized by strong field confinement and narrow spectral features, which are particularly advantageous for refractive-index sensing applications. To evaluate the sensing capabilities, the refractive index of the surrounding medium $n_{medium}$ was systematically varied.

The optical response was extracted via the scattering matrix, where the reflectance R and transmittance T are derived from the squared magnitudes of the $S_{11}$ and $S_{21}$ coefficients:

(2) $$R={\left|S_{11}\right|}^{2},\;T={\left|S_{21}\right|}^{2}.$$

This numerical framework enables a direct correlation between the geometrical parameters of the metasurface and the resulting far-field and near-field optical responses, allowing systematic optimization of the sensing performance.

## 3. Results

**Figure 1:**
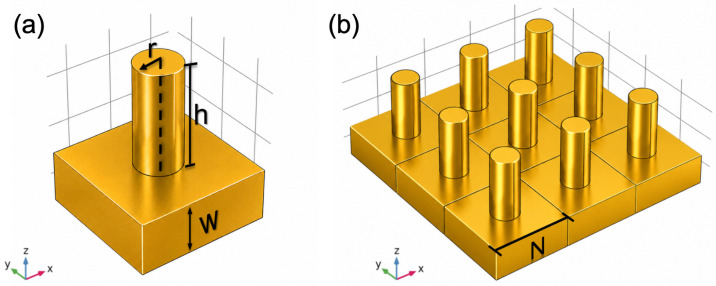
Geometry of the simulated plasmonic sensing platform. (**a**) Unit cell composed of a cylindrical Au nanopillar of radius *r* and height *h* deposited on a continuous Au film of thickness *w*. (**b**) Two-dimensional periodic array obtained by repeating the unit cell with lattice period *N*. The drawings are schematic and are intended to illustrate the geometrical parameters employed in the numerical simulations.

The plasmonic metasurface investigated in this work consists of a periodic array of cylindrical gold pillars placed on top of a continuous gold film, as schematically shown in Figure 1. Each pillar has a radius *r* of 50 nm and a height is *h* of 200 nm, while the thickness of the underlying gold film is denoted as *w* (Figure 1a). The array is periodically repeated in the lateral directions, with the inter-pillar gap in *N* defining the center-to-center separation between adjacent pillars (Figure 1b). The optical response of the metasurface was numerically investigated using FEM simulations under plane wave illumination.

**Figure 2:**
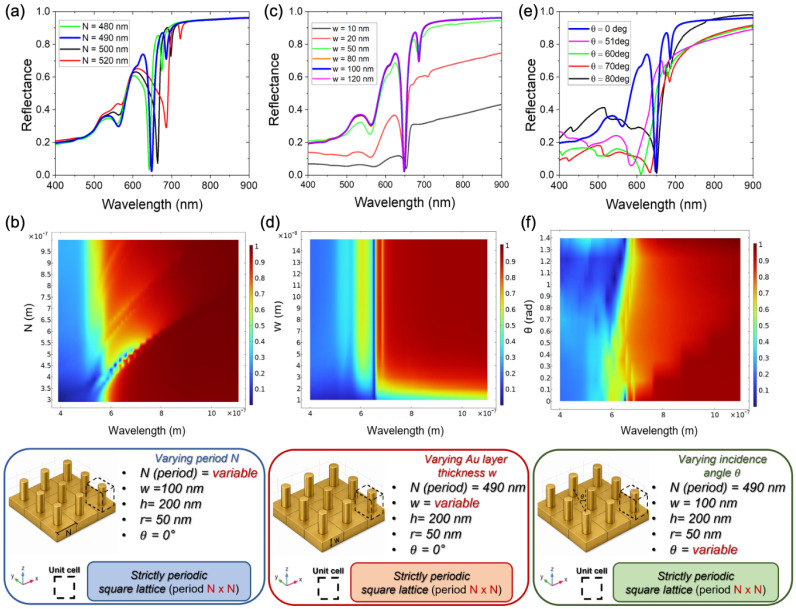
Parametric analysis of the metasurface plasmonic response versus geometry and illumination. (**a**) Simulated reflectance spectra for selected inter-pillar gaps *N*. (**b**) Two-dimensional reflectance map versus wavelength and *N*, highlighting the resonance minima. (**c**) Spectra for different gold-film thicknesses *w*. (**d**) Corresponding two-dimensional map versus wavelength and *w*. (**e**) Spectra at different incidence angles θ, illustrating the angular dependence of the resonance. (**f**) Two-dimensional map versus wavelength and θ.

The influence of the main geometrical parameters and illumination conditions on the plasmonic response of the metasurface was investigated through a systematic parametric study. First, the effect of the inter-pillar gap *N* was analyzed, as this parameter plays a key role in defining the resonance conditions and the collective behavior of the array. The calculated reflection spectra for different gap values, shown in Figure 2a, were obtained by selecting the explored gap range based on the two-dimensional parametric reflection maps reported in Figure 2b, which clearly identify the reflection minima associated with plasmonic resonance excitation. As the inter-pillar gap increases, the resonance wavelength exhibits a systematic red shift, accompanied by variations in spectral contrast.

This behavior can be attributed to the progressive reduction in near-field coupling between the localized surface plasmon modes supported by adjacent pillars, as well as changes in the effective electromagnetic environment experienced by the resonant modes. For small gaps, strong inter-pillar coupling leads to the formation of hybridized plasmonic modes and resonances at shorter wavelengths, whereas increasing the separation weakens the coupling, resulting in a red shift and a gradual stabilization of the resonance position. The observed spectral evolution indicates that the plasmonic response of the metasurface is governed not only by the localized resonances supported by individual nanopillars, but also by collective electromagnetic coupling effects mediated by the periodic arrangement and the underlying metallic film. As the inter-pillar separation increases, the near-field interaction between adjacent pillars progressively decreases, leading to reduced plasmon hybridization and a red shift in the resonance wavelengths.

An optimal inter-pillar gap of 490 nm was identified as providing a spectrally well-defined resonance. Subsequently, the influence of the thickness of the gold film underlying the pillar array was investigated, as this parameter directly affects electromagnetic confinement, reflection efficiency, and resonance quality. The reflection spectra calculated for different gold film thicknesses, reported in Figure 2c, were obtained by considering a thickness range defined based on the corresponding two-dimensional parametric reflection maps shown in Figure 2d. For thinner gold films, the reflection spectra are characterized by reduced spectral contrast, mainly due to increased optical losses and partial transmission through the metal layer. As the film thickness increases, the resonant features become progressively sharper and more pronounced, indicating improved field confinement and reduced leakage into the substrate. An optimal gold film thickness of 100 nm was found to provide the best balance between high reflectivity and well-defined resonances, while further increases do not yield significant improvements, suggesting that beyond this value, the gold layer effectively behaves as an optically opaque reflector. Finally, the dependence of the plasmonic response on the angle of incidence was examined in order to identify the most suitable illumination conditions for sensing applications. The calculated reflection spectra for different angles of incidence, shown in Figure 2e, were obtained by considering an angular range defined on the basis of the two-dimensional parametric reflection maps reported in Figure 2f, which highlight the reflection minima associated with plasmonic resonances. The results demonstrate that normal incidence provides the most efficient and stable excitation of the plasmonic modes, yielding spectrally narrow and high-contrast resonance features. Indeed, under normal-incidence conditions, a more symmetric excitation of the nanostructured array is achieved, preserving the intrinsic spectral sharpness of the hybrid resonance. This configuration ensures stable and well-defined resonances, minimizing angular dispersion effects that could complicate the interpretation of subsequent measurements.

Overall, the parametric analysis reveals that the inter-pillar gap, the thickness of the underlying gold film, and the angle of incidence jointly determine the spectral position, sharpness, and stability of the plasmonic resonances. The optimized configuration, defined by an inter-pillar gap of 490 nm, a gold film thickness of 100 nm, and normal-incidence illumination, emerges as the most suitable design for sensing applications, enabling the excitation of spectrally sharp and high-contrast resonances while ensuring strong near-field confinement at $\lambda_{1}$ = 648 nm and $\lambda_{2}$ = 686 nm, respectively. The simultaneous presence of spectrally narrow resonances and strong spectral contrast suggests the excitation of hybrid plasmonic states resulting from the interplay between localized pillar modes and surface-supported plasmonic modes of the gold film. This hybrid electromagnetic behavior is particularly advantageous for sensing applications, since it combines strong field localization with reduced resonance linewidths, ultimately improving the sensing figure of merit.

**Figure 3:**
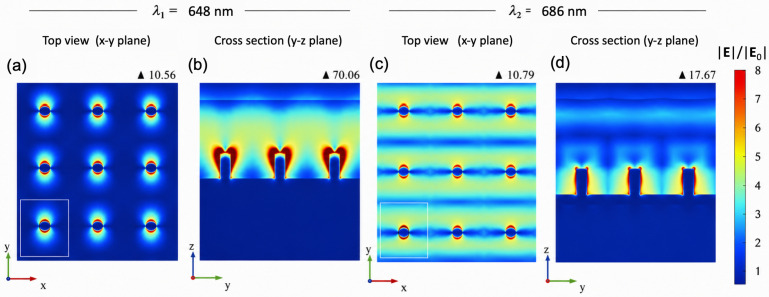
Two-dimensional maps of the normalized electric-field amplitude $\left|E|/\right|E_{0}|$ at the two main resonance wavelengths of the metasurface. (**a**,**b**) First resonance at $\lambda_{1}$ = 648 nm: (**a**) top view in the *x*-*y* plane and (**b**) cross-section in the *y*-*z* plane, showing strong field localization at the pillar edges and in the near-surface region. (**c**,**d**) Second resonance at $\lambda_{2}$ = 686 nm: (**c**) *x*-*y* map and (**d**) *y*-*z* section, highlighting enhanced fields along the metal surface and in the inter-pillar region. Triangles report the maximum value in each panel.

Having identified the optimal geometrical and illumination parameters from the far-field response, we next analyze the near-field distribution at the resonance wavelengths to verify where the electromagnetic energy is actually concentrated and to clarify the modal origin of the observed peaks (Figure 3).

The normalized electric field intensity maps ($\left|E|/\right|E_{0}|$), calculated at the resonance wavelengths $\lambda_{1}$ = 648 nm (Figure 3a) and $\lambda_{2}$ = 686 nm (Figure 3b), corresponding to the two most intense peaks observed in the spectrum, show that even for relatively large gap values, the electromagnetic field remains strongly confined at the pillar edges and in the regions immediately adjacent to the metallic surface, a feature that is crucial for efficient sensing applications. The electric field intensity maps calculated at the resonance wavelengths show distinct features for the two main peaks. The map for the first peak (Figure 3a) reveals a field strongly localized at the edges of the pillars, associated with Localized Surface Plasmons (LSPs) characteristic of individual pillars. In contrast, the map for the second peak (Figure 3b) shows the field concentrated on the metallic surface between the pillars, indicating the presence of Surface Plasmon Polaritons (SPPs) supported by the gold film. The distinct spatial distributions observed for the two resonances indicate different modal origins. The first resonance exhibits highly localized electromagnetic hot-spots concentrated at the pillar edges, characteristic of predominantly localized plasmonic modes. Conversely, the second resonance displays a more spatially extended field distribution along the metallic film, indicating stronger coupling with propagating surface plasmon polaritons. These regions exhibit the highest electromagnetic-field enhancement and therefore provide the strongest interaction with the surrounding dielectric medium. Consequently, local variations in the refractive index efficiently perturb the hybrid LSPR–SPP mode, leading to the pronounced resonance shifts and high sensing performance discussed in the following sections. This pronounced near-field confinement in both configurations ensures efficient interaction with any analyte deposited on or near the metasurface, a critical factor for high-performance sensing.

**Figure 4:**
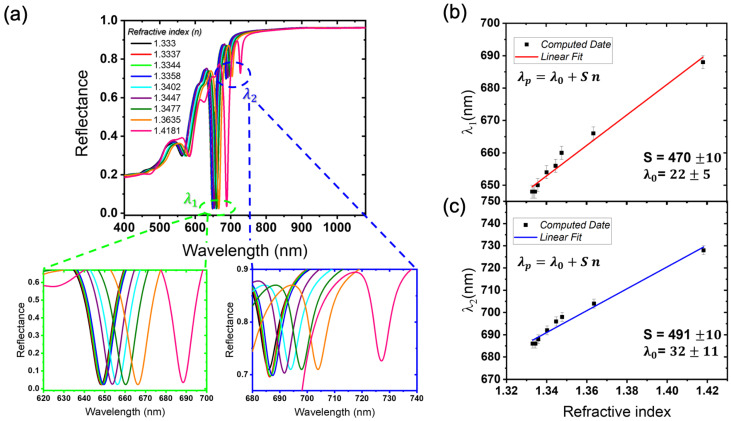
Spectral characterization of the plasmonic metasurface. (**a**) Reflection spectra calculated for different concentrations of glycerol, corresponding to increasing refractive-index values of the surrounding medium. (**b**,**c**) Linear fit of the resonance peak wavelength as a function of refractive index for the two dominant resonant modes ($\lambda_{1}$ and $\lambda_{2}$).

The sensing performance of the optimized plasmonic metasurface was systematically evaluated by monitoring the spectral shifts of its two main resonance peaks in response to controlled variations of the surrounding refractive index $n_{medium}$. To emulate a standard calibration procedure, we numerically modeled aqueous glycerol solutions by assigning to the superstrate the refractive indices corresponding to glycerol concentrations ranging from 0% to 50% (0, 0.5, 1, 2, 5, 8, 10, 20, and 50%). The selected refractive-index interval (approximately 1.333–1.417) is representative of the optical properties of aqueous biological media and several chemical solutions commonly employed for bulk refractive-index sensing, thus providing a realistic benchmark for evaluating the intrinsic sensing performance of the proposed metasurface. The simulated spectra reveal a clear displacement of both resonances, $\lambda_{1}$ and $\lambda_{2}$, toward longer wavelengths with increasing glycerol concentration (Figure 4a). Figure 4b,c report the reflection minima of each peak for all tested concentrations, together with linear fit lines describing the peak shifts as a function of the refractive index. The slopes of these lines directly provide the metasurface sensitivity (S = $\Delta \lambda_{res}$/Δn), which is approximately 470 nm/RIU for the first peak (Figure 4b) and 491 nm/RIU for the second peak (Figure 4c). The figure of merit (FOM), calculated from the initial resonance peak (0% glycerol, pure water) as the ratio between sensitivity and full-width at half maximum (FWHM), is approximately 32 (FWHM = 14.53 nm) for the first peak and 79 (FWHM = 6.23 nm) for the second peak. These values are comparable to, and in the case of the second resonance exceed, typical sensitivities (300–450 nm/RIU) and FOM values (10–60) reported for plasmonic metasurfaces and nanoparticle arrays operating in similar spectral ranges [21,33,34], The enhanced performance observed for the optimized metasurface can be attributed to the combined effect of strong near-field confinement and reduced spectral linewidths enabled by the hybrid plasmonic architecture. In particular, the coexistence of localized and propagating plasmonic modes allows efficient interaction with the surrounding medium while preserving spectrally sharp resonance features, which are essential for high-resolution refractive-index sensing, confirming the competitive performance of the optimized architecture.

## 4. Discussion

The numerical results demonstrate that the optical response of the investigated metasurface arises from the complex interplay between localized plasmonic resonances supported by the gold nanopillars and propagating plasmonic modes sustained by the underlying metallic film. The coexistence of these resonant contributions enables the formation of hybrid electromagnetic modes characterized by strong near-field confinement together with spectrally narrow resonance features. In particular, the optimized pillar-on-film configuration promotes efficient electromagnetic coupling between adjacent plasmonic elements and the metallic substrate, resulting in enhanced field localization and improved interaction with the surrounding dielectric environment.

The parametric analysis revealed that the sensing performance of the metasurface strongly depends on the geometrical coupling conditions and illumination configuration. Variations in the inter-pillar spacing and gold-film thickness significantly influence both the resonance wavelength and spectral linewidth, highlighting the importance of carefully balancing near-field coupling and radiative damping effects. The second resonance mode, associated with stronger surface plasmon polariton contributions, exhibited superior sensing performance, reaching a figure of merit of approximately 79. This behavior can be attributed to the increased spatial overlap between the electromagnetic field and the external dielectric medium, together with the reduced linewidth of the resonance. The obtained sensitivity and FOM values are comparable to, and in some cases exceed, those reported for several plasmonic metasurface sensors operating in similar spectral ranges, confirming the effectiveness of the proposed hybrid architecture for high-resolution refractive-index sensing. In the present work, the nanopillar radius and height were intentionally kept constant to isolate the influence of the lattice period, the Au-film thickness, and the angle of incidence on the hybrid LSPR–SPP response. Nevertheless, these geometrical parameters are also expected to play an important role in determining the sensing performance. Variations in the pillar diameter and height modify the effective metallic volume and the localized surface plasmon resonance, thereby changing its spectral position and its coupling strength with the propagating SPP supported by the underlying Au film. As a consequence, the resonance wavelength, near-field localization, and sensing efficiency can be significantly affected. While moderate increases in pillar dimensions may enhance the interaction between the electromagnetic field and the surrounding dielectric medium, excessively large dimensions are expected to increase radiative damping and ohmic losses, leading to broader resonances and consequently lower FOM values. A systematic optimization of these geometrical parameters represents an interesting extension of the present work and will be addressed in future investigations. The sensing performance obtained for the optimized pillar-on-film architecture can be better appreciated by comparing it with representative plasmonic refractive-index sensors reported in the literature. Table 1 summarizes the main sensing metrics together with the dominant resonance mechanism and the fabrication complexity of representative platforms.

As shown in Table 1, although some diffraction-assisted plasmonic platforms based on surface lattice resonances or Wood-anomaly coupling achieve higher figures of merit, they generally rely on more sophisticated resonance mechanisms and, in several cases, more demanding fabrication strategies. By contrast, the proposed pillar-on-film architecture provides competitive sensing performance while maintaining a relatively simple geometry and enabling a systematic optimization of hybrid LSPR-SPP modes. Although the present study is based on numerical simulations, the investigated geometry is compatible with current nanofabrication approaches such as electron-beam lithography and thin-film deposition techniques commonly employed for plasmonic metasurface fabrication. Nevertheless, practical implementations may be affected by fabrication tolerances, metallic surface roughness, and material losses, which could slightly modify the resonance linewidth and spectral position. In practical implementations, fabrication imperfections such as pillar-diameter variations, height deviations, nonideal sidewall profiles, and surface roughness of the Au film may affect the optical response of the metasurface. These effects are expected to mainly induce resonance broadening, reduced spectral contrast, and small shifts in the resonance wavelength, thereby potentially lowering the FOM. Nevertheless, the systematic parametric analysis reported here indicates that the sensing response is not associated with a single isolated geometrical condition, but rather with a hybrid LSPR–SPP coupling regime that remains accessible within a finite range of structural parameters. Future work will include a dedicated tolerance analysis based on randomized geometrical disorder and experimentally measured roughness profiles. In addition to fabrication-related effects, realistic operating conditions may introduce environmental perturbations, including temperature fluctuations and variations in the optical properties of the surrounding medium. In the present simulations, intrinsic material losses were accounted for by using the experimentally measured dispersive optical constants of gold reported by Johnson and Christy. However, temperature-dependent changes in the refractive index of the analyte solution, substrate, or metal dielectric function were not explicitly included. Such effects may lead to additional resonance shifts or baseline drifts in practical sensing experiments. Therefore, future experimental implementations should include temperature stabilization, reference channels, or self-referencing strategies to distinguish analyte-induced spectral shifts from environmental fluctuations.

Future developments may include the experimental realization of the proposed metasurface, the investigation of ultrathin molecular layers and biomolecular adsorption processes, as well as the extension of the platform toward active, tunable, or chiral sensing configurations. Overall, the presented results provide useful design guidelines for the development of next-generation plasmonic metasurfaces combining strong electromagnetic confinement, narrow spectral features, and high sensing performance. Although the present study focuses on bulk refractive-index sensing, the proposed metasurface is intended for label-free optical detection of analytes that induce local refractive-index variations at the metal surface. Representative applications include the detection of biomolecular interactions (antibody–antigen binding, protein adsorption, DNA hybridization), monitoring of changes in solution composition, and chemical or environmental sensing. In these applications, the adsorption of target molecules modifies the effective refractive index surrounding the nanopillars, producing measurable spectral shifts of the hybrid LSPR–SPP resonance. The present work does not establish a limit of detection for a specific analyte, since this would require modelling the binding kinetics, molecular surface coverage, or a finite biorecognition layer, which are beyond the scope of this numerical study. Instead, the reported sensitivity and figure of merit quantify the intrinsic performance of the plasmonic platform and provide a benchmark for future application-specific sensor implementations.

## 5. Conclusions

In this work, we have designed, optimized, and analyzed a plasmonic metasurface based on gold nanopillars arranged in a periodic array, demonstrating its potential as a versatile, high-performance platform for refractive index sensing. Our parametric studies on pillar geometry, inter-pillar gap, gold film thickness, and illumination conditions allowed us to identify an optimal configuration capable of ensuring strong near-field confinement and extremely sharp resonance peaks. Refractive index sensing simulations, performed by testing different glycerol concentrations, revealed a linear response of the two primary resonance peaks located at 648 nm and 686 nm. Specifically, a sensitivity of approximately 470 nm/RIU was achieved for the first peak and 491 nm/RIU for the second peak. Furthermore, the spectral quality analysis yielded a Figure of Merit (FOM) of 32 for the first resonance and a remarkable value of 79 for the second one. These results confirm the metasurface’s ability to detect infinitesimal variations in the dielectric environment with high precision and reproducibility. Comparative analysis demonstrated that the strong coupling between the plasmonic near-fields and the surrounding medium significantly influences the optical response of the metasurface, allowing for the effective monitoring of even ultrathin molecular layer depositions. Overall, these results demonstrate that the proposed metasurface operates as a highly sensitive platform, whose excellent efficiency stems from accurate structural optimization. The presented design offers promising prospects for practical applications in biomedical, biochemical, and environmental sensing, where accurate and real-time monitoring of the local dielectric environment is essential for identifying trace analytes. This study establishes a solid design principle for the development of next-generation integrated optical sensors, capable of combining high spectral resolution with superior sensitivity.

## Figures and Tables

**Table 1:** Quantitative comparison of representative plasmonic refractive-index sensing platforms reported in the literature. Only values explicitly reported in the original articles are included.

Reference	Structure	Resonance Mechanism	Sensitivity (nm/RIU)	FWHM (nm)	FOM	Fabrication
**This work**	Au pillar-on-film	LSPR–SPP	491	6.23	79	EBL + Au deposition (Moderate)
Shen et al. [19]	Au mushrooms	LSPR–Wood	1015	9.5–12.7	80–108	EBL + multilayer deposition (High)
Li et al. [27]	All-metal array	SLR	501.8	13.5	NR	EBL + metal deposition (Moderate)
Wang et al. [35]	Au grating	Double Fano	470	15	31	FIB milling (High)
Hsiao et al. [36]	Dielectric nanobar	Quasi-BIC	608/612 ^a^	NR	46/85 ^a^	EBL + RIE etching (High)
Ray et al. [37]	Hybrid nanoantenna	Mie–LSPR	208/245	NR	NR	EBL + multilayer deposition (High)

LSPR: localized surface plasmon resonance; SPP: surface plasmon polariton; SLR: surface lattice resonance; EBL: electron-beam lithography; RIE: reactive ion etching; NR: not reported. ^a^ Experimental/numerical values, respectively.

## Data Availability

The data supporting the findings of this study are available from the corresponding author upon reasonable request.

## Abbreviations

The following abbreviations are used in this manuscript:

EWFD	Electromagnetic Waves, Frequency Domain
FEM	Finite Element Method
FOM	Figure of Merit
FWHM	Full Width at Half Maximum
LSP	Localized Surface Plasmon
LSPR	Localized Surface Plasmon Resonance
PBC	Periodic Boundary Condition
PML	Perfectly Matched Layer
RIU	Refractive Index Unit
SPP	Surface Plasmon Polariton
